# Supplementary material for: Chromothripsis during telomere crisis is independent of NHEJ, and consistent with a replicative origin
Source: Genome Res. 2019 May;29(5):737–49. doi: 10.1101/gr.240705.118 (PMC6499312; doi:10.1101/gr.240705.118)
Supplement: Supplemental Material [file supp_gr.240705.118_Supplemental_file_1.zip › contigs/annotated_contigs/DB83/contig.2.DB83_length_616_mean_cov_10.7142857143.docx]

**DB83_length_616_mean_cov_10.7142857143**

```
AAATCACATATGCTTATTTTATATAAAATTAAGTAGTATATGCTATATAATTAGGCAATATATAAATATGAATATGTATCTATCTAAAA
 >chr7:11898920-11899193 + E=1e-152 p=4e-02
ACTAATCTATCTATATAAATTATATATCTTTGTTATTAATAATATACAACAAATATGTCCCGGCCAAGATTATTATTGCCCTTAGGTGG

AATATAAAACTATAAAATATGATGTTAAAGTTCCATTTGTCTAGAATATTTTCCTTTTAAAGGATTAATATCTTCAAGTATTGACCCAT

TTG|TTT|TTATTCTCACAATTATCTAGTGCTGATTTTTATTAAAAGCATCTAGAGTCAGGTGGTTTGATTTGGAACGCTTATTTAAGC
    >chr7:11899890-11900236 + E=2e-196
GCAGATAATAATGTGATAATAATGTGATTATCCAATACATCTAAAACCTTCCATATTATATCCATTTATCTAAATTTTCAGAAATTTAA

AGGTAATCAATCATAACAAGATTCAAGTTCTTGAAACTATAATTTTTATTTCATTGAAAATAATTTAAATAAACCAACTATATATGTAA

ATTTTAAGTACATATGTAAAAATTTAAGTACATTCCATTGTGGATTATTTGCATATTTTAATTGCATGAATAAAGAGACTAAAA
```
